# Sex-Age Related Rumination Behavior of Père David’s Deer under Constraints of Feeding Habitat and Rainfall

**DOI:** 10.1371/journal.pone.0066261

**Published:** 2013-06-18

**Authors:** Zhongqiu Li

**Affiliations:** The State Key Laboratory of Pharmaceutical Biotechnology, School of Life Sciences, Nanjing University, Nanjing, China; University of Tasmania, Australia

## Abstract

Extensive studies have been conducted on the rumination behavior of domestic herbivores. However, studies on wild animals are limited, particularly wild animals with specific ruminating parameters. In this study, Père David’s deer, a previously extirpated species, was observed to analyze the effects of sex-age, feeding habitat, and rainfall on rumination behavior in the Dafeng Nature Reserve, China. Rumination behavior was investigated based on four parameters: proportion of bedding time spent chewing, bolus processing time (s/bolus), chewing frequency (chews/bolus), and chewing rate (chews/s). Results showed that all three factors affect rumination behavior. The extent of their effects varied based on the four rumination parameters. Chewing rate and frequency decreased based on sex–age levels, i.e., from fawns to juvenile female, juvenile male, adult female, stag, and harem holder. Therefore, body size played a major role in shaping rumination behavior. Deer found in grasslands could chew faster compared with deer found in woodlands. This result might be caused by the effects of dietary composition and sunlight intensity. A deer spends a longer time ruminating while bedding during rainy days compared with rainless days to maximize energy and nutrition intake and compensate for the loss of feeding time during rainy days. Therefore, rumination behavior is plastic and is shaped by intrinsic and extrinsic factors.

## Introduction

Rumination behavior is important for food utilization because ruminants mechanically process and reduce forages into small particles by repeated chewing [1], [2]. The comminution of forages increases the surface area of the food available to the bacteria, thereby increasing fiber degradation and utilization in the rumen.

Rumination is sometimes considered a plastic behavior because several intrinsic and extrinsic factors influence chewing parameters [2]–[5]. Body size, which is closely related to sex and age, is the most important factor among all intrinsic factors [2], [6]. The Jarman–Bell principle states that larger animals can feed on poor diets because of their low metabolic requirement to gut capacity ratio. Metabolic requirements are allometrically related to body size, whereas gut capacity or rumen size is isometrically related to body size [7]–[9]. Therefore, larger animals can extract more nutrients than smaller animals because they can keep food in the rumen longer. Compared with larger animals, smaller animals are at a disadvantage when extracting nutrients from low quality diets (i.e. high fiber content). Consequently, animals from different sex–age classes are expected to use different ruminating or chewing strategies to maximize energy and nutrition intake.

Animals exhibit different ruminating patterns depending on the different forages available in habitat [1], [10]. Several characteristics of forage affect the intake of ruminants, particularly the content of vegetable fiber such as cellulose and lignin [11]–[13]. Cellulose is accessible to enzymes that normally digest carbohydrates, whereas lignin has high chemical-degradation resistance. Thus, lignin cannot be easily digested by ruminants [1]. Animals may change their chewing behavior such as increasing bolus processing time or slowing down chewing rates to increase chewing efficiency when feeding in areas with rough forages or digesting lignified and high fibrous roughages.

Environmental factors, including temperature, wind, and precipitation, can also affect rumination [2], [5]. Animals may find difficulty in searching for food under bad weather conditions, such as the presence of low temperatures, strong winds, or heavy precipitation [5]. Bad weather causes animals to change their rumination behavior such as maintaining bedding time and increasing ruminating proportion to compensate for fluctuations in environmental conditions.

Given the importance of rumination behavior, a number of specific chewing parameters, such as time spent chewing while bedding, bolus processing time, number of chews per bolus, and number of chews per min, have been proposed in the study of herbivore rumination [2]. However, considering the difficulty of tracking and observing free-ranging or even semi free-ranging animals [4], most studies were conducted only on domestic ruminants. The goal of this study is to investigate the effects of sex-age, feeding habitat, and rainfall on the abovementioned rumination parameters of semi free-ranging Père David’s deer. The focus of this study is as follows.

### (1) Effects of Body-size Related Characteristics (i.e., Sex-age and Reproductive Status) on Rumination Behavior

According to the Jarman–Bell principle, smaller individuals have higher chewing rates than larger individuals to increase chewing efficiency [7], [8], [14]. Hence, chewing rates should decrease in the following order: fawn, juvenile, adult.

### (2) Effect of Feeding Habitat

Rumination usually occurs shortly after feeding, approximately 10 min for Père David’s deer [15]. Hence, the majority of rumination observations collected from deer in grasslands can be regarded as deer completely feeding on grass. A possibility exists that a deer feeds in woodlands and then rests in grasslands; however, these observations should be rare. Deer ruminating in woodlands are expected to chew slower than deer ruminating in grasslands because a woody diet contains more vegetable fibers, particularly lignin, which is indigestible [1].

### (3) Effect of Rainfall

Bedding is beneficial for saving energy. In the event of rain, deer would increase ruminating proportion when bedding to compensate for the loss of feeding opportunity and increase energy and nutrient intake.

## Methods

### Study Site and Study Subjects

This study was conducted in the Dafeng Père David’s Deer National Nature Reserve (32°59′–33°03′N, 120°47′–120°53′E) in Jiangsu Province, China. The reserve is located on the coast of the Yellow Sea in Eastern China and lies 2 m to 4 m above sea level. The reserve has an annual average temperature of 14.1°C, an average temperature of 0.8 and 27.0°C in January and July, respectively, and 217 frost-free days. The average precipitation is 1068 mm with rain falling mostly between June and September. The Dafeng Nature Reserve consists of three core zones, two of which are enclosed by fences to allow Père David’s deer to range freely. Deer usually forages in grasslands, which are dominated by Chinese pennisetum *(Pennisetum alopecuroides)* and cogongrass *(Imperata cylindrica*), and woodlands, which are dominated by Canadian populus *(Populus canadensis)* and locust *(Robinia pseudoacacia*) [16], [17].

The Père David’s deer is an endangered species with sexual dimorphism. Adult males are approximately 35% heavier than adult females [18], [19]. The mating system is harem polygamous, which means that a strong harem holder dominates the whole harem group and monopolizes nearly all mating opportunities; other stags can only fight or give up mating rights [20], [21]. The rutting season is usually from May to July, and the calving season is from March to May.

The Père David’s deer is a formerly extirpated species in China [22]. A herd of 39 Père David’s deer was reintroduced in the reserve in 1986. After 26 years of conservation and development, the total deer population increased to 1789, thus making this herd the world's largest deer population [23]. The reserve has been implementing a re-wilding program since 1998, and the wild deer population has grown to 182 deer as of 2011. The study was conducted in the first fenced core area, in which a herd of more than 1000 deer live.

### Behavioral Sampling

The study was conducted during the late rutting seasons (July to August) of 2011 and 2012. Observations were conducted along the trails of the first core zone every day to study deer that were bedding. Focal bedding deer were randomly selected and observed by using binoculars (8×56) or a telescope (20∼60×63). The following data were recorded at the beginning of each focal observation: sex-age (fawn, juvenile female, juvenile male, adult female, stag, and harem holder), feeding habitat (grassland and woodland), weather (rainy or rainless), group size and composition (male group, female group, and mixed-sex group), GPS location, date, and time (morning or afternoon). Observations were usually made from a shelter or at a distance of 150 m away from the focal animals to reduce observer effects [24]. Given that the animals were unmarked, sampling the same animal more than once on a given day was unlikely given the large population size.

According to sex–age and reproductive status, deer were classified into six categories: fawns (less than 1 yr, 12.6±1.4 kg, n = 17), juvenile females (between 1 yr to 3 yrs, 100.4±5.7 kg, n = 7), juvenile males (between 1 yr to 4 yrs, 130.1±2.8 kg, n = 8), adult females (older than 3 yrs, 139.0±7.6 kg, n = 22), and stags and harem holders (older than 4 yrs, 184.2±17.2 kg, n = 16) [19]. Although no detailed information was reported regarding the body size of harem holders, they were expected to have larger body sizes than stags because of the competition to monopolize mating rights [21].

During rumination, a bolus of food rises from the rumen; this bolus is then chewed and swallowed (referred to as a bolus cycle) [5]. Focal samples, which consisted of the bedding of randomly selected focal individuals, were observed for 20 min. The 20 min observation of these individuals was only used to analyze the proportion of bedding time spent chewing. Data on deer that ruminated at least five bolus cycles but were observed for less than 20 min were also included in the calculation of bolus processing time and chewing frequency and rate. The data recorded for each individual were the meantime and mean number of chews for a minimum of at least five consecutive bolus cycles. Deer sometimes ruminate while standing. However, this activity is rare; hence, all observations were considered bedding rumination behavior. A bolus usually lasts for approximately 1 min. Thus, each individual was observed for at least 5 min. The four rumination parameters from the observations were quantified as follows: (1) proportion of bedding time spent chewing; (2) mean processing time (sec/bolus); (3) mean chewing frequency (number of chews/bolus); (4) mean chewing rate (chews/sec), which was calculated by dividing mean chews/bolus by mean processing time/bolus.

### Statistical Analysis

A mixed linear model was used to analyze the effects of the possible factors on rumination indices. The majority of samples were collected from big groups (larger than 20 individuals; these groups were usually stable for hours if not disturbed). Thus, group size was not included in the model. For the proportion of bedding time spent chewing, a group ID was included as a random factor, whereas sex-age (fawn, juvenile female, juvenile male, adult female, stag, and harem holder), weather (rainy or rainless), group composition (male, female, and mixed-sex), time of day (morning or afternoon), and second-order interactions were considered fixed factors in the initial model. The feeding habitat was not included as a potential factor because all data were observed from grassland. In the final model, only sex-age and weather were included among the factors because of the non-significance of group composition, time of day, and second-order interactions (P>0.421).

For the other three rumination parameters, a group ID was also included as a random factor. Sex-age (fawn, juvenile female, juvenile male, adult female, stag, and harem holder), feeding habitat (grassland and woodland), weather (rainy or rainless), group composition (male, female, and mixed-sex), time of day (morning or afternoon), and second-order interactions were considered fixed factors in the initial models. However, group composition, time of day, and second-order interactions showed insignificant effects on the rumination parameters (P>0.100); hence, these factors were excluded from the final models. Values were reported by using mean ± standard error with a significance level of P<0.05.

### Ethics Statement

Permission to undertake the field observation was granted by the Management Bureau of Dafeng Père David’s Deer National Nature Reserve (Daming Sun, Director). A telescope was used to observe the rumination behavior of Père David’s deer from a distance of >120 m to minimize observer disturbance. All experiment procedures in this study were approved by the Chinese Wildlife Management Authority, and all animals used in the experiment were managed according to the guidelines of the Chinese Wildlife Management Authority.

## Results

The dataset consisted of 371 focal observations collected during the summer seasons of 2011 and 2012. Among the samples, 42 were from fawns, 43 were from juvenile females, 99 were from juvenile males, 76 were from adult females, 81 were from stags, and 30 were from harem holders. Furthermore, 294 of the 371 observations were collected from grasslands and the rest were from woodlands. The observation days comprised 234 rainy days and 137 rainless days.

A total of 216 observations of 20 min samples were gathered to analyze the proportion of bedding time spent chewing. The final model indicated that both sex-age (F_5, 208_ = 3.023, P = 0.012) and rain (F_1, 208_ = 11.836, P = 0.001) had significant effects on bedding time spent chewing (Table 1). Post hoc test results indicated that fawns spent less time ruminating when bedding compared with all other categories. Deer spent a greater proportion of bedding time ruminating on rainy days (0.702±0.053) than on rainless days (0.468±0.045).

**Table 1:** Proportion of ruminating/bedding of Pére David’s deer with respect to sex-age and rainfall in Dafeng Nature Reserve.

	Rainy day		Rainless day	
	Mean(SE)	N	Mean(SE)	N
Fawn	0.468(0.085)a	7	0.210(0.077)a	17
Juvenile Female	0.884(0.080)b	8	0.625(0.072)b	20
Juvenile Male	0.733(0.056)b	28	0.475(0.055)b	30
Adult Female	0.752(0.062)b	18	0.494(0.055)b	34
Stag	0.721(0.069)b	12	0.462(0.059)b	30
Harem holder	0.664(0.108)b	10	0.405(0.116)ab	2

Same letter in the same row denotes no significant difference at P>0.05.

For chewing frequency, the model showed that sex-age (F_5, 362_ = 26.944, P<0.001) had a significant effect, whereas the effects of feeding habitat (F_1, 362_ = 1.535, P = 0.218) and rain (F_1, 362_ = 1.905, P = 0.170) were insignificant (Table 2). Post hoc tests indicated that deer decreased their chewing frequency in the following order: fawns, juvenile females, juvenile males, adult females, stags, and harem holders. However, no significant difference was observed between stags and harem holders and among adult females, juvenile males, and juvenile females.

**Table 2:** Mean (+SE) of bolus processing time(sec), chews/bolus, chews/sec of Pére David’s deer with respect to sex-age, feeding habitat and rainfall in Dafeng Nature Reserve.

	Bolus processing time				Chews/bolus				Chews/sec				
	Grassland		Woodland		Grassland		Woodland		Grassland		Woodland		
	Mean(SE)	N	Mean(SE)	N	Mean(SE)	N	Mean(SE)	N	Mean(SE)	N	Mean(SE)	N	
Fawn	42.813(0.988)a	34	40.526(1.219)a	8	46.536(1.433)a	34	39.910(3.399)ab	8	1.096(0.326)a	34	0.976(0.058)ab	8	
Juvenile Female	39.594(1.209)ab	36	38.756(1.225)a	7	42.615(1.423)ab	36	40.206(0.918)ab	7	1.087(0.030)a	36	1.046(0.050)a	7	
Juvenile Male	40.093(0.697)ab	76	41.124(1.100)a	23	40.976(0.471)b	76	41.813(0.964)a	23	1.043(0.020)a	76	1.032(0.034)a	23	
Adult Female	40.276(0.618)ab	61	42.306(1.118)a	15	40.068(0.518)bc	61	41.087(1.731)a	15	1.007(0.018)ab	61	0.974(0.034)ab	15	
Stag	38.624(0.660)b	65	41.682(1.615)a	16	35.490(0.610)d	65	34.369(0.979)b	16	0.931(0.018)bc	65	0.836(0.028)b	16	
Harem holder	40.977(1.577)ab	22	39.890(0.966)a	8	34.982(0.654)cd	22	33.848(0.604)b	8	0.868(0.021)c	22	0.851(0.019)ab	8	

Same letter in the same row denotes no significant difference at P>0.05.

For chewing rate, the model indicated that sex-age (F_5, 362_ = 14.867, P<0.001) and feeding habitat (F_1, 362_ = 4.637, P = 0.033) both had significant effects, whereas the effect of rain (F_1, 362_ = 0.122, P = 0.727) was insignificant (Table 2). Post hoc tests indicated that deer increased their chewing rate in the following order: fawns, juvenile females, juvenile males, adult females, stags, and harem males. However, no significant difference was observed between stag and harem males and among adult females, juvenile males, juvenile females, and fawns. Deer found in grasslands chew faster (1.008±0.011) than deer found in woodlands (0.969±0.021).

For bolus processing time, the model indicated insignificant effects for all three factors (sex-age, F_5, 362_ = 1.959, P = 0.084; feeding habitat, F_1, 362_ = 1.414, P = 0.235; rain, F_1, 362_ = 0.731, P = 0.393; Table 2).

## Discussion

Results showed that all three factors, namely, sex-age, feeding habitat, and rain, affected the rumination behavior of Père David’s deer. These factors had varying effects on the rumination parameters. Sex-age influenced almost all four rumination parameters. Feeding habitat only affected chewing frequency and rate, and rain only had a slight effect on the proportion of bedding time spent ruminating.

Sex-age had a significant effect on rumination proportion while bedding, chewing frequency, and chewing rate, as well as a marginal effect on bolus processing time. Numerous studies have addressed the importance of sex and age on shaping rumination behavior [25]–[27]. However, the majority of these studies consider sex–age effects to be closely related to body size [3], [5], [6], [9], [28], which is commonly regarded as the most important variable [2]. When controlling for body size, the effect of sex-age on human chewing effectiveness disappears [29] because body size is isometric with the occlusal surface area of the teeth, which is an important parameter that determines tooth effectiveness [27], [30], [31]. Thus, chewing behavior, which also affects chewing effectiveness [2], compensates for the possible reduction of tooth effectiveness.

Chewing behavior is strongly related to the quantity and quality of food processed [2]. However, food selectivity may also be related to body size. Considering metabolic rates and feeding abilities (e.g., picking out small items), smaller animals tend to select low quantities of high quality food, whereas larger animals forage conversely [8]. When consuming low quality food, a slow chewing rate increases the chewing efficiency of larger animals [14]. Given the difficulties of catching and identifying wild animals, the body weight measurements of each focal individual were not obtained. Therefore, the scaling effect of body weight on rumination parameters could not be analyzed. However, body weight might probably be the most important factor.

Fawns spent approximately 30% on bedding time rumination, which was only approximately half of the other sex-age categories. Fawns can obtain extra (or maybe basic) energy and nutrition from suckling, and the pre-weaning period can last for approximately half a year [32], [33]. No significant difference was observed among other categories, thus indicating that all sex-age levels maintained a certain rumination proportion for nutrition and energy intake when they have grown up.

Although certain studies have reported that reproductive status affects feeding and rumination behavior [3], [34], [35], this effect was not observed among male deer. Harem holders even fast during the peak of rut season [21], [32], which probably affects rumination behavior. However, this study was conducted in July and August when the rut season was nearly finished. Moreover, even if harem holders are larger than other stags, the body size difference might not be significant to change rumination behavior.

Feeding habitat displayed significant effects on chewing rates, thus demonstrating that deer found in grasslands chew faster than deer found in woodlands. The effect of habitat on rumination behavior might be attributed to the different diet composition between grasslands and woodlands. Dominant plants in grasslands include Chinese pennisetum and cogongrass, whereas woodlands are populated by Canadian populus and locust; all of these plants can be utilized by deer [16]. Chewing behavior is influenced by cell toughness, particularly cellulose fibers and lignin in cell walls [1], [10]. Certain studies reported that the relationship between cell toughness and feeding time or chewing effectiveness is uncertain. Spalinger et al. (1996) hypothesized that particle breakdown rate in the rumen should be positively related to the lignin concentration. However, they found that the results they obtained are inconclusive [36]. Nevertheless, this uncertainty may precisely reflect the importance of chewing behavior. When feeding on tough foods, the decision to spend more time on chewing or to slow down chewing rates can consequently increase chewing efficiency. The exact concentrations of cellulose fibers and lignin in the four plants in the two habitats were not measured. However, the two latter trees should contain more vegetable fibers, particularly lignin, even only in their epicormic shoots and branches. High vegetable fibers would lead to a reduction of chewing rate in woodlands. Another possible reason might be the different intensities of sunlight. Many studies have addressed the importance of sunlight on sleeping, rumination, and other behaviors of herbivore [37], [38]. Sheep would change their ruminating patterns under different lighting conditions [38]. When in darkness or low intensity sunlight, animals easily succumb into a rest or relaxed state. The intensity of sunlight and temperature are higher in grasslands than in woodlands. Thus, during the hottest periods of the year, woodlands can provide enough shade for the deer, thus causing them to become relaxed, rested, and ruminate slowly [39], [40].

Weather conditions, including temperature, wind, and rain, affect feeding and rumination behavior [5]. However, unlike the bighorn sheep *(Ovis canadensis)*, which spend less time processing per bolus under rainy conditions [5], Père David’s deer devote more time to rumination while bedding. When raining, the deer have to spend extra energy to search and feed because of low visibility and tough feeding paths. More importantly, rain decreases herbage acceptability because of the palatability effect, which results in a reduction of dry-matter intake [41]. Excessive water in the rumen might not decrease forage intake but would reduce saliva production during the ingestion of low dry-matter forages [42], [43]. Thus, ungulates are usually observed to reduce feeding time, biting rate, and intake per bite on rainy days [5], [41]. For compensation, prolonging rumination time would increase chewing efficiency, thus leading to a higher utilization of forages in the rumen.

In conclusion, rumination behavior could be considered a plastic behavior constrained by sex-age, feeding habitat, rain, and other factors. Among all the abovementioned factors, body size might be the basic characteristic that binds other factors. Rumination should be further explored, such as whether/how deer change their rumination behavior when provided with supplemental food during food-limited seasons (i.e., late November to April) and whether/how harem holders allocate their rumination behavior during the peak of rut season. These studies would help in understanding how Père David’s deer rapidly adapted and recovered in their native land, as well as provide information on how to implement better conservation and management strategies for this species.
